# Single Nanoparticle Detection Using Far-field Emission of Photonic Molecule around the Exceptional Point

**DOI:** 10.1038/srep11912

**Published:** 2015-07-07

**Authors:** Nan Zhang, Shuai Liu, Kaiyang Wang, Zhiyuan Gu, Meng Li, Ningbo Yi, Shumin Xiao, Qinghai Song

**Affiliations:** 1 Integrated Nanoscience Lab, Department of Electrical and Information Engineering, Harbin Institute of Technology, Shenzhen, 518055, China; 2 Department of Material Science and Engineering, Harbin Institute of Technology, Shenzhen, 518055, China; 3 State Key Laboratory of Tunable Laser Technology, Harbin Institute of Technology, Harbin, 158001, China

## Abstract

Highly sensitive, label-free detection methods have important applications in fundamental research and healthcare diagnostics. To date, the detection of single nanoparticles has remained largely dependent on extremely precise spectral measurement, which relies on high-cost equipment. Here, we demonstrate a simple but very nontrivial mechanism for the label-free sizing of nanoparticles using the far-field emission of a photonic molecule (PM) around an exceptional point (EP). By attaching a nanoparticle to a PM around an EP, the main resonant behaviors are strongly disturbed. In addition to typical mode splitting, we find that the far-field pattern of the PM is significantly changed. Taking a heteronuclear diatomic PM as an example, we demonstrate that a single nanoparticle, whose radius is as small as 1 nm to 7 nm, can be simply monitored through the variation of the far-field pattern. Compared with conventional methods, our approach is much easier and does not rely on high-cost equipment. In addition, this research will illuminate new advances in single nanoparticle detection.

The early-stage detection and characterization of single pathogens and viruses are of central importance for disease diagnosis, disease control, environmental monitoring, emergency response, and homeland security^1^. In past decades, a number of methods have been developed to detect cells, DNA, and viruses^2^. However, only a few of them have the ability to efficiently monitor a single virus or single molecule^3^,^4^,^5^,^6^,^7^,^8^,^9^,^10^,^11^,^12^,^13^,^14^. The whispering gallery mode (WGM) resonator has been considered one of the most prominent approaches. The light is trapped by the total internal reflection along the cavity boundary for a long time. and the intensity of the circulating waves inside the WGM cavities are significantly enhanced by several orders of magnitude^15^. Consequently, the interaction between light and matter is improved, and the sensitivity limit is pushed to the single virus level^16^,^17^,^18^. WGM cavity-based label-free detectors can be classified into two categories according to their principles of function. The first one was pioneered by Vollmer and Arnold in 2002^19^, where the bound molecules can be monitored through the shift of resonant wavelengths in a microsphere. This method has quickly attracted considerable research attention and has been successfully utilized to detect single viruses and single molecules^20^,^21^,^22^,^23^. In 2009, Zhu *et al.* demonstrated a different mechanism to detect nanoparticles by measuring mode splitting in an ultrahigh Q microtoroid^24^,^25^,^26^,^27^. This new finding can not only monitor the size of nanoparticles, but it can also better suppresses the noise that the first method suffers from^24^. Soon after, its detection limit was also pushed to the single virus level, and several types of sensors have been developed based on similar physics^28^,^29^,^30^.

In 2014, the concept of EP was introduced in label-free optical detection^31^. The so-called EP is a type of degeneracy in systems that are described by a non-Hermitian Hamiltonian^32^. At such a point, not only the eigenvalues but also the corresponding eigenstates of a non-Hermitian matrix coalesce in parameter space^33^. A number of unique properties have been theoretically predicted and experimentally observed in the past few years^34^,^35^. One prominent example is that the energy splitting is proportional to 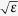 if the EP for two coalescing levels is subjected to a perturbation *ε*. In this sense, the energy splitting at EP will be significantly enhanced when the perturbation strength is sufficiently small. Taking the WGM cavity as an example, Wiersig applied this concept to label-free detection and demonstrated a threefold enhancement in sensitivity, which is extremely important for the improvement of the detection limit^31^. Although all of the above techniques have the ability to detect a single virus or single particle, a tunable diode laser with particularly fine wavelength stability and narrow linewidth must be used in the necessary experiments^2^, significantly impacting the cost and stability of sensors. Therefore, it is vital to find a new mechanism that is independent of changes in the transmission or emission spectrum. A recent report by Sarma *et al.* has provided a very interesting alternative approach^36^. By measuring the changes in the far-field patterns around an EP, the rotation of system can be simply recorded, and the sensitivity is much better than the conventional Sagnac effect, thus demonstrating the significant advantages of far-field direction. Here, we explore the possibility of single nanoparticle detection through the variation of far-field emissions.

## Results and Discussion

### Theoretical analysis

Our findings are also based on the recent developments on EPs. In general, the effective Hamiltonian of a microcavity can be written as a 2 × 2 matrix^33^
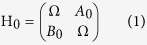
under two-mode approximation and slow varying envelope approximation^37^. Here, Ω is the frequency of unperturbed WGMs, and A_0_, B_0_ are backscattering coupling efficiencies between clockwise (CW) and counter clockwise (CCW) waves. For a system at EP, there is no backscattering in the CW (or CCW) direction even though the opposite backscattering exists. Thus, either A_0_ or B_0_ must be zero. Below, we will take the condition A_0_ > 0 and B_0_ = 0. The case A_0_ = 0 gives similar results.

Once the system is perturbed by an external nanoparticle (or molecule), the effective Hamiltonian is changed to^31^,^32^
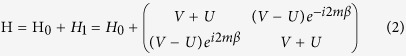
where V, U are the complex frequency shifts for the positive- and negative-parity modes introduced by the external particle. Then, the eigenvalues of the perturbed system can be expressed as
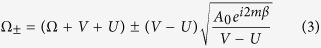
From this equation, it is easy to see that the perturbation of external scatter on the resonances at EP can be much larger than its influence on conventional modes when the perturbation is sufficiently small. This phenomenon is the fundamental basis of the recent proposed microcavity sensor^31^. Rather than the mode splitting, it is more interesting to explore the other resonant properties. Once the resonances are pushed away from the EP, the field distributions of the non-orthogonal mode pairs become partial standing waves inside the cavity. Then, the field is re-distributed inside the cavity, and the modes with different field confinements around the scatter resonate at different frequencies and experience different scatterings^24^. This phenomenon is the fundamental basis of wavelength shift, mode splitting, and changes in the far-field pattern^38^.

The three detection methods have similar sensitivity because they are simply different characteristics of the internal field re-distribution^22^,^28^. However, they show quite different behaviors in practical applications. Compared with the wavelength shift and mode splitting, detecting the far-field pattern is much easier experimentally. Thus, we focus on the influences of external scatter on the far-field pattern. Considering an initial microcavity with unidirectional output at the EPs, it is easy to imagine the degradation of the unidirectionality when the system is perturbed by an external nanoparticle. Two scattering effects contribute to the far-field pattern. First, the scattering of a nanoparticle usually gives multiple directional emissions without considering the collimation of the microcavity. Then, the unidirectional emission will be “diluted” by the scattering. Second, the scattering can affect the percentage of energy that reaches the initial leaky channels that form the directional output. Consequently, the unidirectional output is also changed. Therefore, in addition to the very precise spectral measurement, the degradation of directionality in the far-field pattern can be a potential way of sizing the nanoparticle.

It is worth noting the essential role of the EP here. As mentioned above, the resonances around EP are non-orthogonal mode pairs and copropagate in either the CW or CCW direction. Then, the modes receive similar scattering from the external nanoparticle, and their unidirectional outputs are both degraded. In contrast, once the resonances are away from EP, interference between the CW and CCW components generates standing waves. The anti-symmetric mode has near-zero field distribution around the nanoparticle and is almost independent of small perturbations. Thus, the total degradation of the unidirectional emission is smeared out.

### Numerical simulation

**Figure 1:**
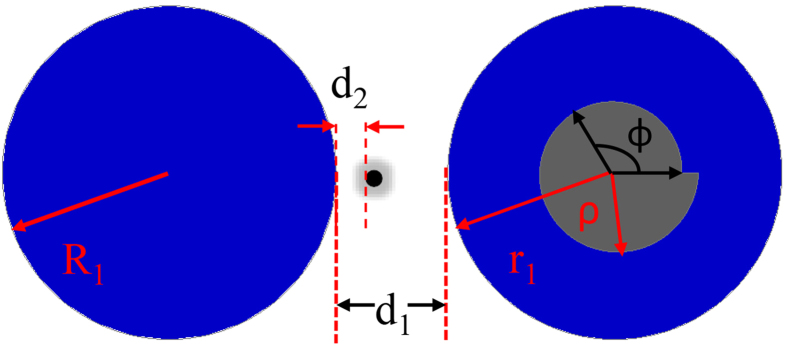
The schematic picture of photonic molecule. The radiuses of left and right circles are R_1_ and r_1_, respectively. The inner boundary of annular ring is 
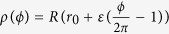
 and parameters are r_0_ = 0.56, ε = 0.16. R_1_ = 0.9985R, r_1_ = R, d_1_ = 0.8R, β_1_ = 0. The radius of nanoparticle is r_2_ positioned at the Azimuthal position β_2_ (here β_2_ = 0). The separation distance between nanoparticle and circular cavity is d_2_ = 0.015R.

Below, we take a heteronuclear diatomic PM as an example to illustrate the above analysis. Compared with the single cavity, PM supports modes with narrow linewidths, wide mode spacing, and greatly enhanced sensitivity to the changes in the dielectric constant of their environment^39^,^40^. Importantly, the heteronuclear diatomic PM can be an optimal platform to generate the combination of high Q factor, high chirality, and unidirectional output^41^. Here, the chirality is different from the conventional chiral media and can be defined by the different components in the CW and CCW directions, following the equation
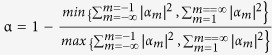
31,32. As illustrated in Fig. 1, the PM consists of a circular cavity and an annular cavity. The inner boundary of the annular cavity is a spiral shape that is defined in polar coordinates as
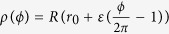
. Below, we focus on the mode around kR = 4.3436, which corresponds to a wavelength of approximately 1.46 μm when R = 1 (fixed below). The target nanoparticle is positioned at the azimuthal position *β*_*j*_, and the separation distance between the nanoparticle and the circular cavity is d_2_. The radius of the nanoparticle is *r*_*2*_. Note that because the wavelength is much larger than the size of the scatter, the precise shape is not important in our system.

**Figure 2:**
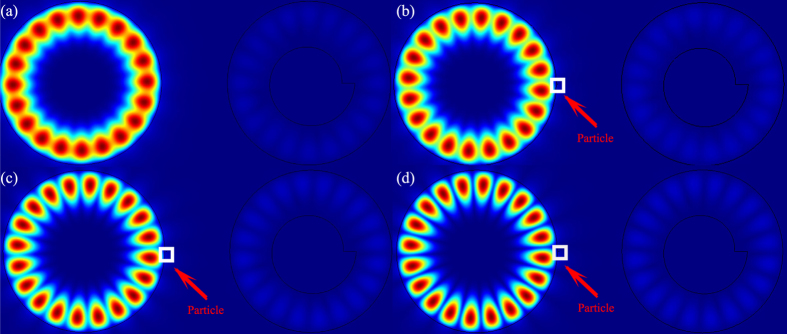
Near-field distribution of resonance mode at KR~4.3436–0.001086i. (**a**) Without target particle. A target particle at β_2_ = 0 with the radius r_2_ = 2 nm (**b**), r_2_ = 3 nm (**c**), and r_2_ = 4 nm (**d**).

Then, we numerically calculate the resonant properties of TE (transverse electric) modes by solving Maxwell’s equations using the effective index approximation^42^. The refractive indices of the PM and nanoparticle are 3.3 (for GaAs or silicon) and 1.5 (for polystyrene). The external environment is set at n = 1, following the experiment presented in Ref. 24. Figure 2 shows the field patterns of modes with the azimuthal number *m* *=* *10* and the radial number *l* *=* *1*. Without external perturbation, the field distribution inside the circular cavity is quite uniform, which reflects the fact that the modes near EP are traveling waves rather than standing waves. When a target particle is placed near the circular cavity, the field distributions gradually change. As shown in Fig. 2(b), a visible nodal line starts to appear and becomes very apparent when the particle size is *r*_*2*_ *=* *4* *nm* (see Fig. 2(d)), which means that the modes inside the cavity turn from propagating waves to partially standing waves and that the resonant modes have been gradually pushed away from the EP. The standing waves are formed by the interference between the CW propagating waves and the scattered waves along the CCW direction. As mentioned above, the interference redistributes the field. Because the modes are still close to the EP, it is difficult to separate the symmetric mode (SM) and anti-symmetric mode (ASM), as is done in conventional studies^24^. Below, we define SM and ASM as the modes with relatively stronger and weaker field distributions inside and around the nanoparticle, respectively.

**Figure 3:**
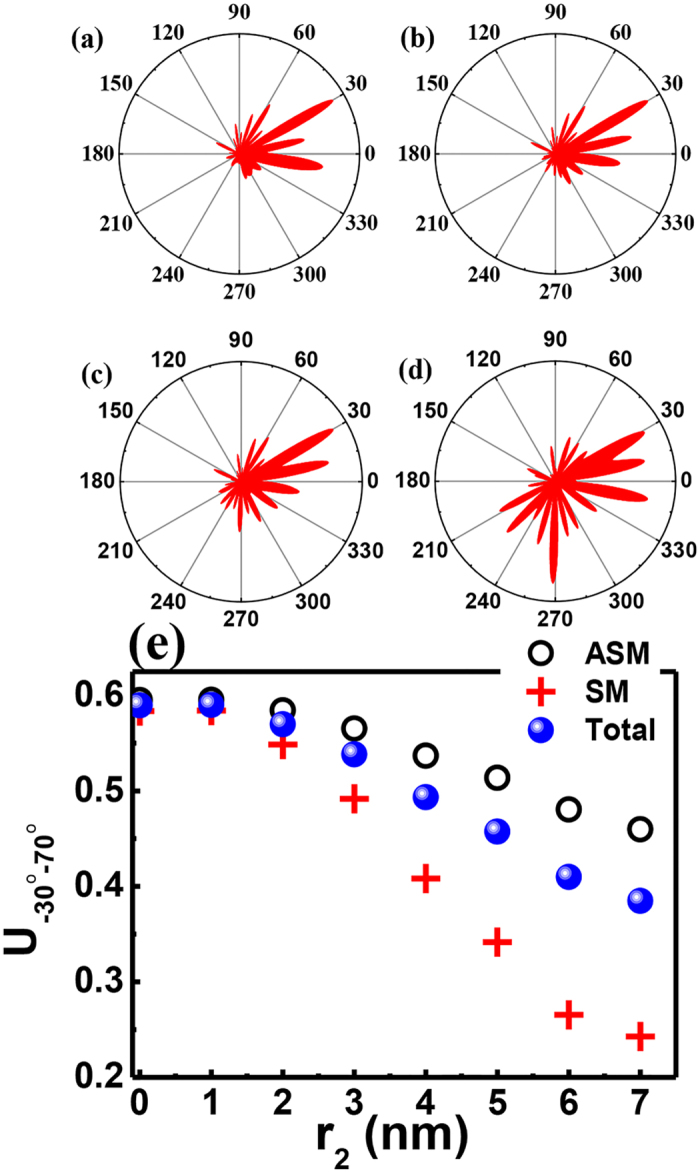
The far-field patterns of the same resonance as Fig. 2. The radius of nanoparticle is r_2_ = 0 nm (**a**), 2 nm (**b**), 4 nm (**c**), and 7 nm (**d**). (**e**) shows the fraction total emitted light in ϕ_FF_ = −30° to ϕ_FF_ = 70° (blue dots) and its components with ASM (circles) and SM (crosses) symmetries as a function of r_2_.

We then examine the far-field pattern of the resonant mode as a function of particle size to test our analysis. Partial results are shown in Fig. 3. Without the external nanoparticle, the PM shows clear unidirectional emission in the approximate angle range −30^o^ −70^o^ (see Fig. 3(a)). Once the external particle is attached to the circular cavity, the far-field pattern gradually changes. With an increase in particle size from 1 nm to 7 nm, we can observe that the unidirectional emission within the angle range −15°− 45° reduces and the emissions approximately 210°–300° increase quickly. When the particle size r_2_ is larger than 6 nm, the far-field pattern turns to nearly bi-directional emissions (see the example in Fig. 3(d)).

To quantitatively characterize the change in the far-field pattern, we have defined a measure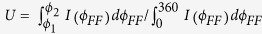 to represent the percentage of energy within the angle range ϕ_1_ < ϕ_FF_ <is the angular distributionϕ_2_, where I(ϕ_FF_) is the angular distribution of the far-field pattern. All of the results are summarized as blue dots in Fig. 3(e), where ϕ_1_ = −30°, ϕ_2_ = 70°. As r_2_ increases from 0 to 7 nm, the U_−30°−70°_ factor quickly decreases from 0.6 to 0.4, which indicates that the unidirectional emission is partially destroyed. Thus, it is easy to determine that the external scatter can affect the far-field pattern. The detailed calculations show that the far-field patterns of both SM and ASM are reduced, although the SM modes with stronger field distributions inside the nanoparticle (see the open circles and crosses in Fig. 3(e)) become stronger. These entire phenomena are consistent with the above analysis.

**Figure 4:**
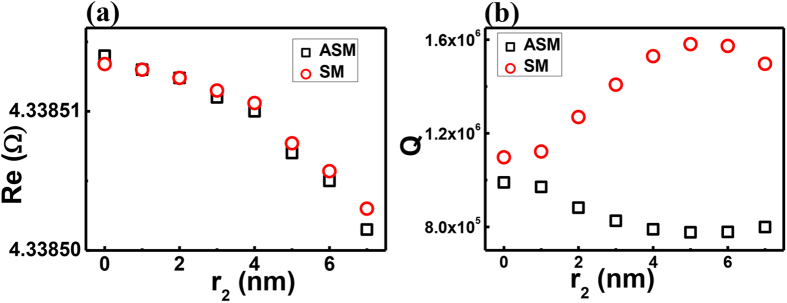
The resonant frequencies and Q factors of SM (red circles) and ASM (squares) as a function of nanoparticle size.

The important question is how such directionality is affected by the target particle. The most intuitive expectation is that the unidirectional emission of the initial mode is “diluted” by the multiple directional emissions caused by the scattering at the nanoparticle. To verify this possibility, we have studied the resonant behaviors of the hybrid modes in PM. As shown in Fig. 4, with increasing particle size, both SM and ASM shift to smaller frequencies, similar to conventional studies. However, the behaviors of Q factors are quite different. The Q factor of ASM, which receives less influence from the nanoparticle, is slightly reduced, by approximately 20%. The Q factor of SM first increases and then decreases slightly. The response of SM’s Q factor, particularly the increase in the Q factor, contradicts the conventional understanding. Following the intuitive configuration, strongly scattering light to the far field induces a larger loss and thus reduces the Q factors. Thus, the scattering to the far field is not the main mechanism for the degradation of the U factor.

In addition to scattering electro-magnetic waves to the far field, the nanoparticle can influence the percentage of waves that enter the annular cavity. Stronger scattering prevents the evanescent waves from reaching the annular cavity. As discussed in Ref. 41, the unidirectional output is completely formed by the scattering at the notch in the annular cavity of the PM. Once the energy that enters the annular cavity is reduced, the unidirectional emission is disturbed simultaneously. When the size of the nanoparticle is small, its scattering loss is smaller than the scattering at the notch (the Q factor of the PM without nanoparticle is far smaller than a circular cavity that is perturbed by a tiny nanoparticle). Then, the Q factor is primarily determined by the loss in the annular cavity. Once the percentage of energy that can reach the notch is decreased, it is easy to imagine that the loss is reduced and the Q factor is increased. Consequently, the SM mode, which is strongly affected by the nanoparticle should have a higher Q factor than the ASM, which is close to the nodes. Meanwhile, because the nanoparticle only scatters the ASM slightly, the changes in Q are correspondingly smaller. This information is exactly what we have observed in Figs 3(e) and 4(b), where the stronger degradation in U_−30°−70°_ and increase in the Q factor both occur in the SM. As the nanoparticle’s radius continues to grow, the loss induced by the scattering at the nanoparticle continues to increase and becomes comparable with the leakage inside the annular ring. This effect contributes to a reduction in the Q factor and to strong disturbance in the far-field pattern. This result is also consistent with the results with larger r_2_ in Fig. 4(b).

**Figure 5:**
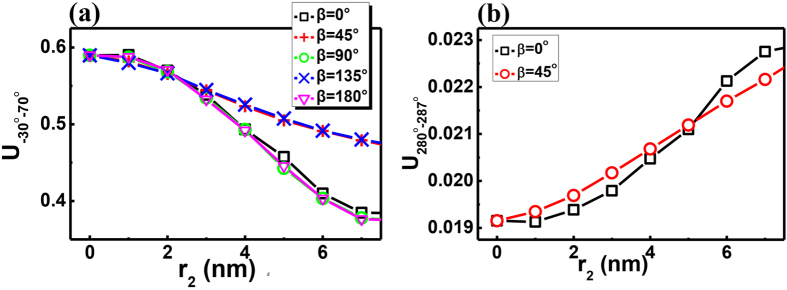
(**a**) The U_−30°−70°_ as a function of radius of nanoparticle at different positions. (**b**) The corresponding directional output in angle range 280–287 degree as a function of r_2_. With these two curves, the position dependence of detection can be eliminated.

The above analysis and numerical results show that the variation in the far-field pattern might be a way to detect nanoparticles. This technique does not require the ultrahigh Q factor and extremely fine spectral resolution that are necessary for the conventional method. There is one remaining technical challenge in detection. As shown in Eq. (3), the influence of the external nanoparticle clearly shows position dependence. Figure 5(a) demonstrates the corresponding numerical results at different angles. We find that the U_−30°−70°_ factors with the target particle at the azimuthal angle β = 0, π/2, π are almost the same, and U_−30°−70°_ with the particle at the azimuthal angle β = π/4 and 3π/4 are also very close. However, the curves at β = 0 and β = π/4 are quite different, especially in the range r_2_ > 3 nm. The U_−30°−70°_ factors at other β values fall between them. This type of deviation is consistent with Eq. (3) and results from the interaction between scattered waves from the nanoparticle and annular cavity^32^. Because the azimuthal number is 10, β = 0, π/2, π give the same value in Eq. (3), as do β = π/4 and 3π/4. Therefore, the deviation induces unexpected inaccuracy. For example, the U_−30°−70°_ factors with (β = 0, r_2_ = 4 nm) and (β = π/4, r_2_ = 6 nm) are very close in Fig. 5(a).

Interestingly, this inaccuracy can also be eliminated by measuring the far-field pattern. In addition to the unidirectional emission formed by the notch, there are some waves that deviate from the resonant orbit and quickly leak out. The percentage of such waves becomes larger with increasing size of the target particle. Because these leakages have a different mechanism from the unidirectional emission formed by the notch, their dependences on the particle size are also different. Figure 5(b) shows an example emission in the angle range 280°–290°. Though the U_−30°−70°_ factors seem to be very close when particles with different sizes are positioned at 0° and 45° in Fig. 5(a), the difference between their U_280°−290°_ factors is more than an order of magnitude greater. Thus, the slight difference in Fig. 5(a) can be enhanced and distinguished using this additional information.

## Conclusion

In summary, we have proposed a new mechanism to detect single particles using the far-field pattern. We have demonstrated that the far-field pattern is highly sensitive to the target nanoparticle. With the increasing radius of the target particle, the unidirectional emission gradually transitions to multiple directional outputs in the far-field pattern. For a resonant wavelength of approximately 1.45 μm, we have confirmed that individual polystyrene nanoparticles with radii between 1 nm to 7 nm in an air environment were detectable. This result indicates that our method has great potential for detecting single biological protein molecules. Moreover, this optical detection method does not require ultra-sensitive or high-precision equipment, which makes single-particle detection simpler and more cost-effective. We also note that the observed phenomena are not limited to the reported mode in this article. Monotonic reduction in the U_−30°−70°_ factor has also been observed in a PM that is directly formed by a circular cavity and a spiral cavity.

## Method

Because the thicknesses of microdisks are much smaller than their in-plane dimensions, microdisks are usually treated as two-dimensional objects by applying effective refractive indices *n*. Then, the wave equations for transverse electric (TE, E is in plane) polarized modes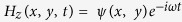 can be replaced by the scalar wave equation
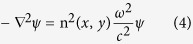
where ω is the angular frequency and *c* is the speed of light in vacuum.

We numerically computed the TE polarized resonances by solving the above equation using FEM with the RF module in COMSOL Myultiphusics 3.5a. The cavity shape is defined using AutoCAD and imported into the software. The outgoing waves are absorbed by a perfect matched layer at the far-field, which leads to quasibound states with complex eigenfrequencies (ω). The Q factor of resonance is determined by 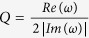, and the far-field patterns are obtained by calculating the outgoing power flow at the position of 20R away from the cavity^43^.

## Additional Information

**How to cite this article**: Zhang, N. *et al.* Single Nanoparticle Detection Using Far-field Emission of Photonic Molecule around the Exceptional Point. *Sci. Rep.* **5**, 11912; doi: 10.1038/srep11912 (2015).
